# Bayesian Inference for Source Reconstruction: A Real-World Application

**DOI:** 10.1155/2014/507634

**Published:** 2014-09-25

**Authors:** Eugene Yee, Ian Hoffman, Kurt Ungar

**Affiliations:** ^1^Defence Research and Development Canada, Suffield Research Centre, P.O. Box 4000 Stn Main, Medicine Hat, AB, Canada T1A 8K6; ^2^Health Canada, Radiation Protection Bureau, 775 Brookfield Road, A.L. 6302A, Ottawa, ON, Canada K1A 1C1

## Abstract

This paper applies a Bayesian probabilistic inferential methodology for the reconstruction of the location and emission rate from an actual contaminant source (emission from the Chalk River Laboratories medical isotope production facility) using a small number of activity concentration measurements of a noble gas (Xenon-133) obtained from three stations that form part of the International Monitoring System radionuclide network. The sampling of the resulting posterior distribution of the source parameters is undertaken using a very efficient Markov chain Monte Carlo technique that utilizes a multiple-try differential evolution adaptive Metropolis algorithm with an archive of past states. It is shown that the principal difficulty in the reconstruction lay in the correct specification of the model errors (both scale and structure) for use in the Bayesian inferential methodology. In this context, two different measurement models for incorporation of the model error of the predicted concentrations are considered. The performance of both of these measurement models with respect to their accuracy and precision in the recovery of the source parameters is compared and contrasted.

## 1. Introduction

In recent years, there have been remarkable advances in sensor technology for the environmental monitoring and surveillance of chemical, biological, or radiological agents released into the atmosphere, either deliberately or accidentally. Two examples of operational monitoring sensor networks are the deployment of biological sensor arrays by the Department of Homeland Security in various cities across the United States as part of the BioWatch program [1] and the global network of radionuclide sensors that form part of the International Monitoring System deployed under the auspices of the Comprehensive Nuclear-Test-Ban Treaty [2]. In parallel with this development, there has been also significant theoretical and computational progress in the modeling and simulation of the transport and dispersion of materials released into the atmosphere, involving ever more accurate representations of the whole complex of processes on the entire spectrum of scales responsible for the dispersion of windborne materials (from the microscale turbulent motions in the atmospheric boundary layer that determine the short-range dispersion to the larger-scale motions associated with various weather systems within the whole global atmosphere that determine the long-range transport).

Lying at the nexus of these two developments is the requirement for ever more sophisticated statistical analysis tools that can be used to fuse the information embodied in the measurements of agent concentration provided by the sensors and in the predictions of these concentrations provided by the atmospheric dispersion model(s). This data-driven sensor-modeling paradigm will enable the characterization of the unknown source following event detection by the network of sensors (inverse problem), which in turn can lead to a greatly improved situational awareness and to a more informed decision-making process for response.

Inverse source modeling is a notoriously ill-posed problem in the following sense: (1) the sparse sampling (in space) of and the presence of multiple sampling times for the concentration in a dispersing cloud of contaminant may lead to difficulties of nonuniqueness in the source reconstruction (namely, there may be many source configurations that are consistent with the limited number of concentration data); (2) the physical processes of mixing and diffusion of material in the atmosphere, with continuing dilution, smooth the concentration field leading to a progressive loss of high-frequency information that could potentially lead to an instability in the source reconstruction; and (3) actual measurements of concentration by sensors and predictions of concentrations by atmospheric dispersion model(s) are never exact (owing to measurement and model errors, resp.) which exacerbates the nonuniqueness associated with incomplete concentration observations and the instability associated with the intrinsic smoothing of the concentration field by the natural mixing processes in the atmosphere.

To overcome the problems of nonuniqueness and instability in the inverse source modeling problem, the most popular approach is regularization which emphasizes the selection of a “well-behaved” source configuration from the possibly infinite set of such configurations that are consistent with the limited number of noisy concentration measurements. In this approach, the idea is to find a source configuration that minimizes (optimizes) a penalty functional that involves two independent terms, namely, a regularization functional (source model norm) that imposes some constraint on the solution and a misfit functional (also some form of norm) that quantifies the discrepancy between the measured and predicted concentrations. For the application of regularization to inverse source modeling problems, see, for example, Robertson and Persson [3], Robertson and Langner [4], Thomson et al. [5], Bocquet [6], and Allen et al. [7].

Regularization provides a single “optimal” selection for the unknown source distribution (*deterministic* solution) with no rigorous quantification of the uncertainty in this particular selection. An alternative to obtaining a single optimal source distribution is to characterize an ensemble of source distributions using the Bayesian statistical paradigm (providing a fully* probabilistic* solution allowing the uncertainty in the source reconstruction to be rigorously assessed). The Bayesian inferential methodology for source reconstruction provides fully probabilistic information on all the parameters used to describe the unknown source distribution. The probabilistic approach using a Bayesian inferential scheme for source reconstruction has been developed by Keats et al. [8], Chow et al. [9], Senocak et al. [10], Yee et al. [11], and Yee [12–15]. The primary limiting factor in the application of the Bayesian inferential methodology for source reconstruction is computational and, as a result, it has not been as widely used as the regularization methodology.

Most of the applications of the Bayesian inferential methodology for source reconstruction have used high-quality concentration data from well-designed atmospheric dispersion experiments to validate the schema. The objective of this paper is to use concentration data obtained from an operational network of sensors (more specifically from a very small subset of the global network of radionuclide sensors that form part of the International Monitoring System) to provide a real-world test of source reconstruction based on the Bayesian statistical paradigm applied to long-range atmospheric transport on a continental or hemispheric scale. It will be demonstrated that one of the key problems that need to be properly addressed for the successful application of Bayesian inference for source reconstruction when using operational concentration data is the “correct” specification of the expected model errors arising from the putative accuracy of a long-range forecast (or, alternatively, reanalysis) of meteorological fields and the concomitant prediction of material dispersion based on this forecast or reanalysis (which can result in significant model errors with a complex structure in the predicted concentrations required for the source inversion).

## 2. Bayesian Probability Theory

In a remarkable paper, Cox [16] demonstrated that probability theory, when interpreted as logic, is the only calculus that conforms to a consistent theory of inference. This demonstration provides the firm logical basis for asserting that probability calculus is* the* unique quantitative theory of inference. More specifically, the cornerstone of logical inference is embodied by Bayes' theorem which itself is nothing more than the product law of probability calculus (or Bayesian probability theory):

(1) $$\begin{array}{c} p\left(\theta \;|\;I\right)p\left(d\;|\;\theta ,I\right)=p\left(d\;|\;I\right)p\left(\theta \;|\;d,I\right). \end{array}$$

In (1), *p*(·) denotes the probability of a proposition or hypothesis, *“*∣*”* denotes “conditional upon” or “given that”, ***θ*** denotes a set of parameters of a model under consideration, **d** denotes the data used for the inference, and *I* denotes the background assumptions or contextual information available in the problem. More specifically, as applied to the problem of source reconstruction, ***θ*** can be identified with the set of parameters used to characterize the source distribution (e.g., location and emission rate); **d** can be associated with the measured concentration data; and *I* corresponds to the contextual information that is relevant to the source reconstruction problem (e.g., reanalyzed or forecast meteorology, atmospheric dispersion model used to define the source-receptor relationship).

The input quantities for Bayesian inference are on the left-hand side of (1) and are as follows: *p*(***θ***∣*I*) is the prior probability for a hypothesis about the values of the source parameter vector ***θ*** which encodes our state of knowledge about these parameters before the receipt of the concentration data **d** and *p*(**d**∣***θ***, *I*) is the likelihood function and is considered to be a function of ***θ*** for fixed data **d**. The likelihood function incorporates the information provided by the measured concentration data **d** into the inferential scheme.

The output quantities provided by the Bayesian inference are on the right-hand side of (1) and are as follows: *p*(**d**∣*I*) is referred to as the evidence or global likelihood and *p*(***θ***∣**d**, *I*) is the posterior probability for the hypothesis about the values of the source parameter vector ***θ***, evaluated in light of the additional information provided by the measured concentration data **d**. The evidence is given by the following multidimensional integral over the source parameter space [cf. (1)]:

(2) $$\begin{array}{c} p\left(d\;|\;I\right)=\int p\left(\theta \;|\;I\right)p\left(d\;|\;\theta ,I\right)d\theta , \end{array}$$

which ensures the proper normalization of the posterior distribution *p*(***θ***∣**d**, *I*) (namely, the condition that the integral of the posterior distribution over its domain of definition is unity). In the context of the determination of the plausible values for the source parameter vector (parameter estimation problem), it should be noted that the evidence is simply a normalization constant that is* independent* of the source parameter vector and, as a consequence, can therefore be ignored. However, it should be* stressed* that the evidence assumes a central role in the problem of model selection as discussed in Yee [15] and, in this context, this quantity cannot be ignored as in the parameter estimation problem considered herein.

In view of the fact that the *p*(**d**∣*I*) is merely a normalization constant in the context of the source estimation problem, the key output of the Bayesian inference methodology in this case is the posterior probability *p*(***θ***∣**d**, *I*) which embodies all the information about the unknown source parameters ***θ***. In particular, *p*(***θ***∣**d**, *I*) provides the full solution for the inverse source determination (or source term estimation) problem. Determining this quantity requires assigning appropriate functional forms for the two input quantities for Bayesian inference: (1) the prior probability *p*(**d**∣*I*) and (2) the likelihood function *p*(**d**∣***θ***, *I*).

### 2.1. Assignment of the Likelihood Function

To assign a specific functional form for the likelihood function, one needs to relate the source parameters ***θ*** to the available concentration data **d**. In this sense, the likelihood function defines the probabilistic model of how the concentration data were generated. Towards this purpose, if the concentration data acquired (by a sensor) at the space-time point (**x**_*J*_, *t*_*J*_) is represented by *d*(**x**_*J*_, *t*_*J*_), then the concentration data and the source parameters ***θ*** are assumed to be related by

(3) dJ≡d(xJ,tJ)=C−(θ;xJ,tJ)+e(xJ,tJ)≡C−J(θ)+eJ, J=1,2,…,N,    

where *N* is the total number of measured concentration data. In (3), ${\overset{-}{C}}_{J}(\theta )$ is the modeled (predicted) mean concentration at the *J*th space-time point. The predicted concentration is determined using an atmospheric dispersion model for a source distribution characterized by the parameter vector ***θ***.

The error (discrepancy) between the measured concentration *d*_*J*_ and the predicted concentration ${\overset{-}{C}}_{J}(\theta )$ is represented symbolically in (3) by *e*_*J*_. In the problem considered in this paper, there are two major contributions to the error *e*_*J*_, namely, the observation or instrument error in the measured concentration *d*_*J*_ arising from the noise inherent in the sensor and the model error in the determination of the predicted concentration ${\overset{-}{C}}_{J}(\theta )$. Of these two errors, the model error is by far the most dominant contribution to the total error *e*_*J*_ and is also the most difficult to characterize.

In our current application, the model error arises from three primary sources. These are as follows:

1. uncertainties in the representation of various physical processes in the dispersion model;
2. uncertainties in the input meteorological fields (initial and boundary conditions) used to “drive” the dispersion model (either numerical weather prediction uncertainties if these fields are obtained as a forecast or data assimilation uncertainties for the state of the atmosphere if these fields are obtained through a reanalysis);
3. uncertainties in the numerical solution of the model equations that characterize the dispersion model which comprise both discretization (including representativity) errors and statistical model errors, the latter of which arises from using necessarily a finite number of “marked” fluid particles to estimate the mean concentration field in the case of a Lagrangian stochastic model of dispersion.

As a consequence of the complexity in structure of the error *e*_*J*_ [cf. (3)] for our current application, it is extremely difficult (if not insuperable) to specify* a priori* an exact value *σ*_*J*_ for the standard deviation of *e*_*J*_ (*J* = 1,2,…, *N*). If the standard deviation *σ*_*J*_ of *e*_*J*_ was exactly known, then it can be shown by application of the principle of maximum entropy that a Gaussian distribution of the form

(4) $$\begin{array}{c} p\left(d\;|\;\theta ,I\right)=\frac{1}{\prod_{J=1}^{N}\sqrt{2\pi }\sigma_{J}}\exp\left(-\frac{1}{2}\chi^{2}\left(\theta \right)\right), \end{array}$$

where

(5) $$\begin{array}{c} \chi^{2}\left(\theta \right)\equiv \overset{N}{\underset{J=1}{\sum }}{\left(\frac{d_{J}-{\overset{-}{C}}_{J}\left(\theta \right)}{\sigma_{J}}\right)}^{2}, \end{array}$$

would be the most conservative choice for the direct probability (or likelihood) of the concentration data **d** ≡ (*d*_1_, *d*_2_,…, *d*_*N*_) [17].

Because *σ*_*J*_ is not known* a priori*, it is useful to characterize the uncertainty in the specification of *σ*_*J*_ with a probability distribution. Following Yee [15], we choose this probability distribution to be an inverse gamma distribution with the following form:

(6) φ(σJ ∣ sJ,α,β)=2αβΓ(β)(sJσJ)2βexp⁡(−αsJ2σJ2)1σJ,  mmmmmmmmmmmmmmmmmmJ=1,2,…,N.

Here, Γ(*x*) denotes the gamma function, *α* and *β* are scale and shape parameters of the inverse gamma distribution, and *s*_*J*_ is the quoted (nominal) estimate for the true but unknown standard deviation *σ*_*J*_. The values for the hyperparameters *α* and *β* are chosen as *α* = *π* ^−1^ and *β* = 1 following the rationale described in Yee [15].

The likelihood function in (4) and (5) depends on the error standard deviations *σ*_*J*_ (*J* = 1,2,…, *N*) which are generally unknown. To remove these unwanted parameters (nuisance parameters), we can multiply the likelihood given in (4) by the (assigned) probability distribution for each of the error standard deviations embodied in (6) and integrate the result with respect to the unwanted parameters (error standard deviations) to give an integrated likelihood function with the following form [15]:

(7) p(d ∣ θ,s,α,β) =∫p(d ∣ θ,I)∏J=1Nφ(σJ ∣ sJ,α,β)dσ =∏J=1NαβΓ(β+1/2)2πsJΓ(β)[α+(dJ−C−J(θ))22sJ2]−β−1/2.

In (7), **s** ≡ (*s*_1_, *s*_2_,…, *s*_*N*_) is the vector of estimated (quoted) standard deviations for the error *e*_*J*_ (*J* = 1,2,…, *N*) and *d* **σ** ≡ *dσ*_1_ *dσ*_2_ … *dσ*_*N*_.

To be more specific with respect to the source parameter vector ***θ***, a source distribution *S* with the following form is considered in this paper:

(8) $$\begin{array}{c} S\left(x\right)=Q_{s}\delta \left(x-x_{s}\right), \end{array}$$

where *δ*(·) is the Dirac delta function. Equation (8) describes a (continuous) point source located at the vector position **x**_*s*_ that is emitting contaminant at a constant emission rate *Q*_*s*_. The parameters describing this source distribution can be assembled into a source parameter vector given by ***θ*** ≡ (**x**_*s*_, *Q*_*s*_).

### 2.2. Assignment of the Prior

To assign the prior probability for the source parameters ***θ***, it is necessary to state explicitly what is known about these parameters. Firstly, it is assumed that the source parameters are logically independent with the result that the prior distribution *p*(***θ***∣*I*) factorizes as follows:

(9) $$\begin{array}{c} p\left(\theta \;|\;I\right)=p\left(x_{s}\;|\;I\right)p\left(Q_{s}\;|\;I\right). \end{array}$$

Secondly, the source location and emission rate are known* a priori* to be bounded. In particular, it is assumed that the location **x**_*s*_ of the source is contained in some spatial region *𝒟* ⊂ *ℝ* ^3^. Furthermore, the emission rate *Q*_*s*_ is assumed to be bounded by *Q*_min⁡_ < *Q*_*s*_ < *Q*_max⁡_ where *Q*_min⁡_ and *Q*_max⁡_ are the lower and upper bounds for the emission rate, respectively. Finally, if nothing else is known about these parameters except for the bounds, then application of the principle of maximum entropy to our state of knowledge concerning the source parameters results in the assignment of a uniform prior distribution for these parameters, so

(10) $$\begin{array}{c} p\left(\theta \;|\;I\right)\propto I_{D}\left(x_{s}\right)I_{\left(Q_{\min},Q_{\max}\right)}\left(Q_{s}\right), \end{array}$$

where *𝕀*_*A*_(*x*) denotes the indicator function for set *A* defined as *𝕀*_*A*_(*x*) = 1 if *x* ∈ *A* and *𝕀*_*A*_(*x*) = 0 if *x* ∉ *A*.  

### 2.3. Posterior Distribution: The Output

The key output of the Bayesian inference is the posterior distribution *p*(***θ***∣**d**, *I*) which can be obtained by combining the assignments for the likelihood function and the prior distribution given by (7) and (10), respectively, to give [cf. (1)]

(11) p(θ ∣ d,s,α,β,I)  ∝ID(xs)I(Qmin⁡,Qmax⁡)(Qs)  ×∏J=1NαβΓ(β+1/2)2πsJΓ(β)  ×[α+(dJ−C−J(θ))2/(2sJ2)]−β−1/2.

Note that the quantities **s**, *α*, and *β* have been added explicitly to the posterior probability of the source parameters ***θ*** in (11) to indicate that these quantities are known (namely, they are provided* a priori* by the user, in addition to the measured concentration data **d**).

## 3. Computational Aspects

There are two computational problems that need to be addressed before the Bayesian inferential methodology for source reconstruction can be applied to practical real-world applications; namely, (1) that Bayesian inversion of concentration data requires a fast and efficient technique for the determination of the source-receptor relationship (namely, for the fast computation of ${\overset{-}{C}}_{J}(\theta )$ for a given hypothesis about the source parameters ***θ***) and (2) methodology for the efficient sampling of the posterior distribution *p*(***θ***∣**d**, **s**, *α*, *β*, *I*).

To address the first problem, Keats et al. [8] and Yee et al. [11] demonstrated that the use of a receptor-oriented representation for the source-receptor relationship (rather than the more usual source-oriented representation) enabled the efficient computation of the predicted concentration at a fixed receptor location associated with the exploration of a large number of source parameter hypotheses required in the simulation-based Bayesian inference methodology. For the application reported herein, a backward Lagrangian stochastic (LS) model for long-range transport was used to determine the adjoint concentration field *C* ^*^ over the northern hemisphere.

The backward LS model employed here is an operational model used by the Canadian Meteorological Centre to support both Canadian Treaty monitoring and the various mandates of the Provisional Technical Secretariat of the Comprehensive Nuclear-Test-Ban Treaty Organization, including event analysis by member states. The backward LS model for the determination of *C* ^*^ was “driven” by reanalyzed meteorological fields that were obtained at a relatively low temporal and spatial resolution, namely, at a temporal resolution of 6 h (rate of the data assimilation) with a core spatial resolution of 0.5° on a geographical latitude and longitude coordinate system. The backward LS model was run retrospectively using these reanalyzed meteorological fields, providing *C* ^*^ fields with a temporal resolution of 3 hours over a period of 14 days prior to the commencement of the sampling for a particular activity concentration measurement.

It should be noted that, with the source distribution *S* given by (8), the predicted concentration at a receptor location **x**_*J*_ and time *t*_*J*_ can be easily determined from the following relationship once the adjoint concentration field *C* ^*^ has been computed for this location and time:

(12) C−J(θ)≡C−(θ;xJ,tJ)=∫−∞tJdt′∫Ddx′C∗(x′,t′ ∣ xJ,tJ)S(x′)≡〈C∗ ∣ S〉(xJ,tJ),

where *C* ^*^(**x**′, *t*′∣**x**_*J*_, *t*_*J*_) is the adjoint concentration at space-time point (**x**′, *t*′) associated with the sensor concentration datum measured at location **x**_*J*_ and time *t*_*J*_. In (12), *𝒟* is a volume in space that contains the source. The reader is referred to Marchuk [18] and Le Dimet and Talagrand [19] for a more detailed explanation of the adjoint formulation in a more general context.

Note that the predicted mean concentration ${\overset{-}{C}}_{J}(\theta )$ “seen” by a sensor for a given hypothesis about the source parameters ***θ*** can be rapidly computed by simply evaluating the inner (or scalar) product 〈*C* ^*^∣*S*〉 of the adjoint concentration *C* ^*^ and the source distribution *S* corresponding to the given hypothesis. In other words, the predicted concentration ${\overset{-}{C}}_{J}$ is obtained from a mathematical model (backward LS model for dispersion) by evaluation of the bounded linear functionals ${\overset{-}{C}}_{J}=\langle C_{J}^{*}|S\rangle$ for *J* = 1,2,…, *N* where *C*_J_^*^ denotes the adjoint concentration field obtained at the sensor space-time point (**x**_*J*_, *t*_*J*_) and where *S* corresponds to a given hypothesis about the source. In applied mathematics, the elements *C*_J_^*^ are referred to usually as representers. More specifically, if we substitute (8) into (12), the model (predicted) concentration ${\overset{-}{C}}_{J}(\theta )$ “seen” by the sensor at location **x**_*J*_ and time *t*_*J*_ is given explicitly by

(13) $$\begin{array}{c} {\overset{-}{C}}_{J}\left(\theta \right)=Q_{s}\overset{t_{J}}{\underset{-\infty }{\int }}C^{*}\left(x_{s},t^{\prime }\;|\;x_{J},t_{J}\right)dt^{\prime }. \end{array}$$

To address the second computational problem mentioned above, we apply a Markov chain Monte Carlo (MCMC) algorithm for the efficient sampling of the posterior distribution of the source parameters (see Gilks et al. [20], Gelman et al. [21], Savchuk and Tsokos [22], and Yuen [23]). In general, MCMC algorithms generate a Markov chain whose stationary distribution coincides exactly with the target probability distribution that we are trying to sample from (which in our case is the posterior distribution *p*(***θ***∣**d**, **s**, *α*, *β*, *I*) given in (11)). The Metropolis-Hastings (M-H) algorithm [20, 21] forms the underlying basis for MCMC sampling and it is perhaps not too surprising that the M-H algorithm has become almost synonymous with MCMC sampling. Indeed, most of the algorithms for MCMC sampling reported in the literature [20–23] can be interpreted as either special cases or extensions of the basic M-H algorithm.

For the current study, we use a multiple-try differential evolution adaptive Metropolis algorithm with sampling from an archive of past states which is referred to as MT-DREAM_(ZS)_. The details of this MCMC sampling algorithm are described by Laloy and Vrugt [24], but, for completeness, we will briefly summarize the main components of this algorithm. In particular, only the relevant details of the algorithm that are required for the interpretation of the results in this paper are emphasized. Firstly, MT-DREAM_(ZS)_ samples from an archive of past states to generate the candidate points (proposals) for each of the individual Markov chains that are used to explore the target posterior distribution. Secondly, as already alluded to here, the algorithm utilizes multiple (different) Markov chains that are run simultaneously in parallel. These multiple chains employ a self-adaptive randomized subspace sampling of difference vectors from the archive of past states to generate new candidate points in these chains. As part of the randomized subspace sampling strategy, each element of the candidate points for the parallel proposals is updated (accepted) in accordance with a binomial scheme (Bernoulli trial) with a crossover probability *p*_*s*_; otherwise, the proposed element retains its previous (old) value. This multiple-chain approach automatically adjusts or adapts the scale and orientation of the proposal function. Thirdly, a snooker step update of the state with an adaptive step size is included with a fixed (albeit small) probability in order to improve the mixing efficiency of the algorithm for exploration of the hypothesis space. The updated states from the various parallel Markov chains are periodically stored in the archive of past states after every *K* (*K* ≥ 1) updates.

Fourthly, to further improve the efficiency of the sampling, MT-DREAM_(ZS)_ incorporates a multiple-try Metropolis (MTM) approach proposed initially by Liu et al. [25]. The basic idea underpinning the MTM approach is as follows: longer range candidate moves are rarely accepted, but if multiple points are proposed for these longer range moves then the acceptance probability will be increased. The MTM algorithm is applied individually to each of the different Markov chains used in the MT-DREAM_(ZS)_, involving generating *K* draws using the randomized subspace sampling procedure for each chain, choosing one of these draws (proposals) as the reference point, and generating a new set of (*K* − 1) draws with respect to this reference point using the randomized subspace sampling strategy. The acceptance rule is simply the Metropolis-Hasting acceptance probability applied to the sequence of proposals that comprise the MTM schema.

Finally, to determine if the multiple Markov chains used in MT-DREAM_(ZS)_ have achieved stationarity (namely, have converged to the stationary distribution associated with the chains), the Gelman and Rubin [26] convergence diagnostic $\hat{R}$ is computed for each dimension of each chain using the last 50% of the samples of the chain. This simple convergence statistic determines whether a chain has achieved stationarity by comparing the variance within each chain and the variance between chains (interchain variance).

## 4. Example: A Real-World Application

The International Monitoring System (IMS) consists of a comprehensive network of seismic, hydroacoustic, infrasound, and radionuclide sensors as part of the verification regime of the Comprehensive Nuclear-Test-Ban Treaty (CTBT) which bans nuclear explosions. A subset of the IMS is the subnetwork of radionuclide gamma detectors/particle filters for the measurement of the activity concentration for various radionuclides (e.g., particulate or aerosol species such as Cs-137 and I-131 and/or noble gases such as Xe-133). The IMS radionuclide network will (eventually) have 80 monitoring stations worldwide for the measurement of the activity concentration for particulate/aerosol radioactive species, of which at least 40 stations would also have the capability to measure the activity concentration of noble gases [2]. The stations provide 12 or 24 h averaged activity concentrations of the various radionuclides depending on the technology used.

We applied the proposed source reconstruction methodology to some (albeit limited) measurements of activity concentrations by a very small subset of the IMS radionuclide network. The release event consisted of the stack emissions from Chalk River Laboratories (CRL) which houses an international production facility for medical radioisotopes. Chalk River Laboratories, which at a latitude of 46.15°N and at a longitude of −77.37°E, is located about 180 km northwest of the city of Ottawa, Ontario. A characterization of the weekly stack emissions of Xe-133 from the CRL medical isotope production facility over a 5-year period yielded a median daily emission of about 24 TBq (or, equivalently, an emission rate of about 1.0 × 10^12^ Bq h^−1^).

**Figure 1:**
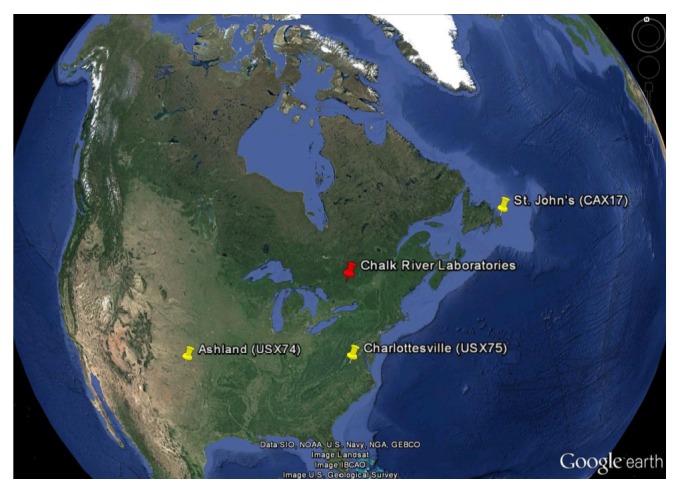
Locations of the three sampling stations from the International Monitoring System radionuclide network used for the source reconstruction. The release location of the Xe-133 tracer source was at Chalk River Laboratories.

Xe-133 activity concentrations were detected and measured by three sampling sites in North America. These three sampling sites are shown in Figure 1. The three sites displayed are part of the noble gas monitoring network of the CTBT verification regime. More specifically, the three stations are as follows: CAX17 (St. John's, Newfoundland, at latitude 47.59°N and longitude -52.74°E); USX75 (Charlottesville, Virginia, at latitude 38.0°N and longitude -78.4°E); and USX74 (Ashland, Kansas, at latitude 31.17°N and longitude -99.77°E).

At each monitoring site, one of two different monitoring technologies is employed to measure radioxenon. The St. John's site has a Système de Prélèvements et d'Analyse en Ligne d'Air pour Quantifier le Xénon (SPALAX) high-resolution gamma system operating on a 24 h sample collection period, while the remaining sites have a Swedish Automatic Unit for Noble Gas Acquisition (SAUNA) beta-gamma coincidence system with a 12 h sample collection period. Both systems employ activated charcoal to remove xenon from the air for radioxenon analysis. After the measurement process is complete, the xenon sample can be stored in an archive bottle for optional remeasurement either on-site or in an off-site laboratory. The stable xenon volume collected varies approximately from 2 mL to 8 mL depending on the technology used, but both technologies have a roughly equivalent Xe-133 sensitivity of approximately 0.2 mBq m^−3^.

The monitoring data for activity concentrations of Xe-133 were obtained from a single month (December 2011). The three monitoring stations used for this study were known to be operating normally during this period of time. For the purposes of source reconstruction, we used 36 of these concentration samples extracted from the three sampling sites. The concentration time series data at each station were blocked averaged over a temporal duration of 3 days to give 8 concentration data *d*_*J*_ that were used as the input for the source reconstruction methodology (namely, *N* = 8 for this example). It should be noted that predictions for the temporally averaged concentration data were subject to smaller statistical sampling errors (for a fixed number of marked fluid particles used in the backward LS model), which is the primary reason for aggregating the 12 or 24 h measurements in this manner. As mentioned earlier, reanalyzed meteorological fields were used to “drive” an operational backward LS model to determine *C* ^*^ which were utilized subsequently for the fast calculation of the predicted concentration ${\overset{-}{C}}_{J}(\theta )$ for an arbitrary hypothesis ***θ*** about the source.

In addition to the measured activity concentration data *d*_*J*_ and the predicted activity concentration data ${\overset{-}{C}}_{J}(\theta )$, it is also necessary to provide an estimate for the noise error variance *s*_*J*_^2^ [cf. (7)]. The estimate for the noise error variance includes two major contributions; namely, (1) an estimate for the sensor error sampling variance *s*_*e*,*J*_^2^ in the measurement of *d*_*J*_ and (2) an estimate for the model error variance *s*_*m*,*J*_^2^ in the prediction of ${\overset{-}{C}}_{J}(\theta )$. These estimates for the two contributions to the noise error variance were added in quadrature to give *s*_*J*_^2^ = *s*_*e*,*J*_^2^ + *s*_*m*,*J*_^2^. Very good estimates for the sensor error standard deviation (or square root of the variance) were provided for the expected precision in the measurements of the activity concentration at each of the three sampling stations. In contrast, no estimates for the precision in the predicted concentrations were available. In light of this, the model error standard deviation was assumed (rather arbitrarily) to be 50% of the predicted concentration ${\overset{-}{C}}_{J}(\theta )$.

For our example, the emission source is assumed to be at or near ground level so that the height of the source is not of any interest in the reconstruction. As a result, the unknown location parameters for the source are its longitudinal (*x*_*s*_) and latitudinal (*y*_*s*_) positions, so ***θ*** = (*x*_*s*_, *y*_*s*_, *Q*_*s*_). The proposed stochastic sampling algorithm constructs an initial population for the archive of past states by sampling from the prior distribution *p*(***θ***∣*I*) (see (10)). The hyperparameters defining the prior distribution *p*(***θ***∣*I*) are chosen as follows: *Q*_min⁡_ = 1.0 × 10^15^ *μ*Bq h^−1^, *Q*_max⁡_ = 1.0 × 10^20^ *μ*Bq h^−1^ which prescribes the lower and upper bounds for the source emission rate and *𝒟* = (−125°E, −45°E)×(25°N, 75°N) which defines the prior bounds for the location (*x*_*s*_, *y*_*s*_) of the source. It is noted that *𝒟* encloses the North American continent.

**Figure 2:**
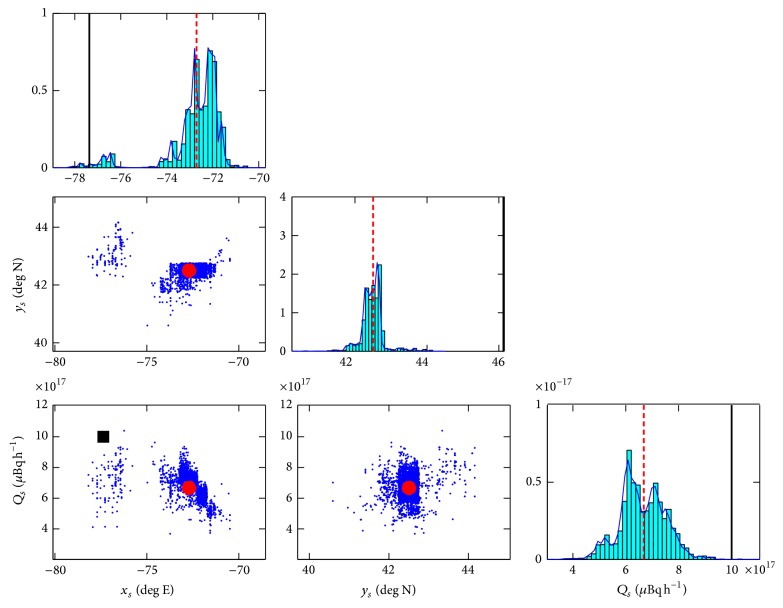
Univariate (diagonal) and bivariate (off-diagonal) marginal posterior distributions of the source parameters; namely, longitudinal position *x*_*s*_, latitudinal position *y*_*s*_, and emission rate *Q*_*s*_. The true parameter values are shown by a solid square or a solid vertical line and the best estimates of the parameter values are represented as a solid circle or a dashed vertical line.

The samples of ***θ*** drawn from the posterior distribution *p*(***θ***∣**d**, **s**, *α*, *β*, *I*) (cf. (11)) using the MT-DREAM_(ZS)_ sampling algorithm were used to determine the characteristics (location and emission rate) of the source. Estimates of these source parameters were obtained directly from the multiple chains of samples generated by the MCMC algorithm after convergence of the chains as determined by the Gelman-Rubin $\hat{R}$ statistic.  Figure 2 exhibits the univariate (diagonal) and bivariate (off-diagonal) marginal posterior distributions for the source parameters. For the univariate marginal posterior distribution of a parameter, the solid vertical line delineates the true value of the parameter, and the dashed vertical line corresponds to the best estimate of the parameter obtained as the posterior mean. Similarly, for the bivariate marginal posterior distribution of various combinations of parameters, the solid square marks the position of the true source parameter values and the solid circle exhibits the best estimate of the true source parameter values obtained as the posterior mean. It should be noted that the axes limits are chosen to display the regions of the highest probability in the marginal posterior probability distributions for the parameters and, in certain cases, these regions do not contain the true parameter values (with the result that some of the panels in Figure 2 do not contain either the solid square or solid vertical line representing the true parameter values).

**Figure 3:**
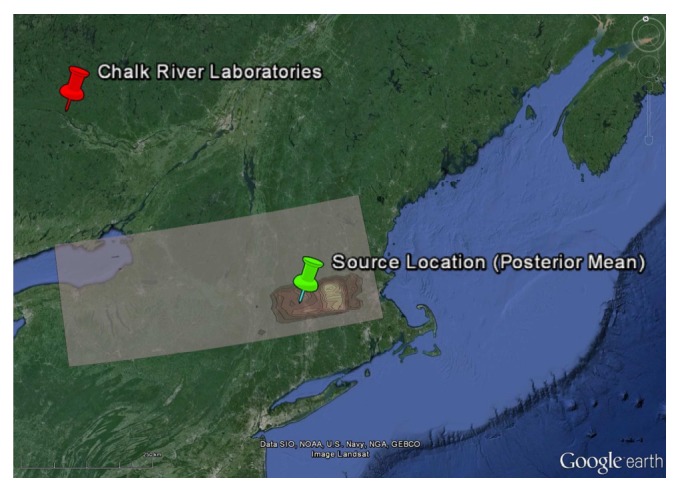
Two-dimensional marginal posterior distribution of the source location georeferenced on a Google Earth image.

The posterior mean, posterior standard deviation, and lower and upper bounds for the 95% highest posterior distribution (HPD) interval (the* p*% HPD (or credible) interval is that interval that contains the parameter with a* p*% (posterior) probability, with the lower and upper bounds of the interval specified such that the probability density within the interval is everywhere larger than that outside it) for the reconstructed source parameters are summarized in Table 1. It is noted that the accuracy in the reconstruction of the source parameters is fairly good for both the location (considering the fact that the reconstruction was undertaken on a continental scale) and the emission rate. More specifically, the distance between the true source location and the best estimate of the source location (obtained as the posterior mean) is only about 572 km (see Figure 3) and the best estimate of the emission rate (obtained again as the posterior mean) is within 33% of the true emission rate.

Even so, a perusal of Table 1 indicates that the precision in the estimates of both the source location and the emission rate is poorly determined. Indeed, it should be noted that the 95% HPD intervals for longitudinal/latitudinal positions and the emission rate of the reconstructed source do not contain the true source parameters. This defect in the source reconstruction can be attributed to the difficulties in providing good estimates for the model errors in the prediction of ${\overset{-}{C}}_{J}(\theta )$. It is evident that the inability to provide good estimates for the model errors (which in principle can be quite significant in our example) can lead to a loss of power in the source reconstruction and may mask important features of the measured activity concentration data.

Given the complexity in the heteroskedastic variance of the model error in our application, the specification of the prior information on the structure of the model error is extremely difficult. In light of these seemingly insuperable difficulties, we consider an alternative measurement model to that introduced earlier in (3). To this purpose, let us now focus on the following alternative measurement model:

(14) $$\begin{array}{c} d_{J}=m_{J}{\overset{-}{C}}_{J}\left(\theta \right)+n_{J},\;\;\;J=1,2,\ldots ,N, \end{array}$$

where *m*_*J*_ are unknown multipliers (scale factors) that are applied to the predicted concentration ${\overset{-}{C}}_{J}(\theta )$ in order to compensate for the model uncertainty. It is important to note that, in (14), the *n*_*J*_ term represents* only* the measurement error in the activity concentration *d*_*J*_. The model errors incurred in the predicted concentration ${\overset{-}{C}}_{J}(\theta )$ are compensated by the introduction of the multiplier *m*_*J*_.

For the alternative measurement model, the multipliers *m*_*J*_ (*J* = 1,2,…, *N*) are unknown parameters and need to be estimated in addition to the source parameters. Let us denote the source parameters in this alternative model by ***θ*** ^*s*^ ≡ (**x**_*s*_, *Q*_*s*_) and by ***θ*** ^*m*^ ≡ (*m*_1_, *m*_2_,…, *m*_*N*_) all other relevant parameters (multipliers in our example). These latter parameters are usually referred to as nuisance parameters. Both sets of parameters define the parameter vector as ***θ*** = (***θ*** ^*s*^, ***θ*** ^*m*^). The likelihood function for the alternative measurement model is still given by (7) with the implicit understanding that the estimated noise variances *s*_*J*_^2^ now* only* include the measurement error contribution (namely, *s*_*J*_^2^ = *s*_*e*,*J*_^2^). As already noted above, this contribution to the uncertainty is well characterized in our current application (implying that the prior uncertainty in the measurement errors can be specified correctly). For the alternative measurement model, one requires also the specification of the prior distributions for the multipliers (nuisance parameters). Towards this objective, uniform priors defined over the range from *m*_min⁡_ to *m*_max⁡_ will be adopted as priors for the multipliers. In view of this, the prior distribution for the alternative measurement model replaces (10) by the following assignment:

(15) $$\begin{array}{c} p\left(\theta \;|\;I\right)\propto I_{D}\left(x_{s}\right)I_{\left(Q_{\min},Q_{\max}\right)}\left(Q_{s}\right)\times \overset{N}{\underset{J=1}{\prod }}I_{\left(m_{\min},m_{\max}\right)}\left(m_{J}\right). \end{array}$$

**Figure 4:**
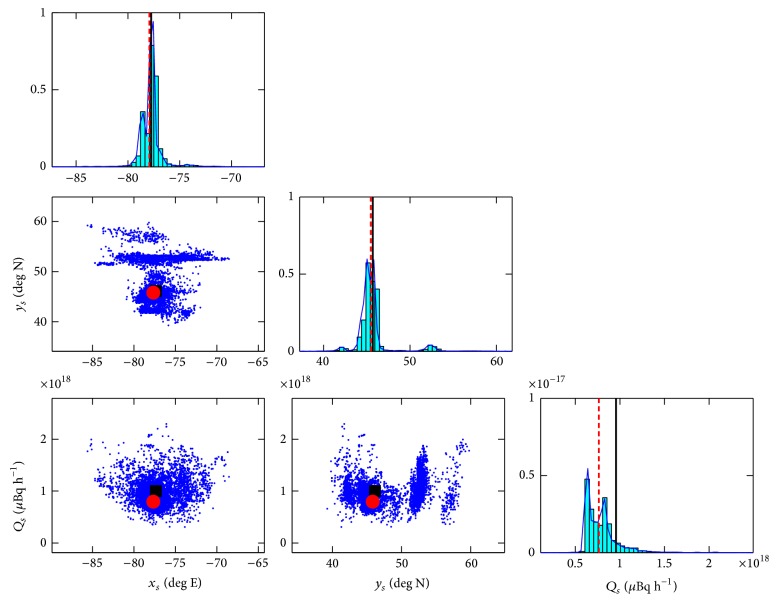
Univariate (diagonal) and bivariate (off-diagonal) marginal posterior distributions of the source parameters; namely, longitudinal position *x*_*s*_, latitudinal position *y*_*s*_, and emission rate *Q*_*s*_. The true parameter values are shown by a solid square or a solid vertical line and the best estimates of the parameter values are represented as a solid circle or a dashed vertical line. The reconstruction was obtained using the alternative measurement model which employs multipliers to compensate for the unknown model error structure.

Using the new measurement model and the modified likelihood function and prior distribution, we applied the MT-DREAM_(ZS)_ algorithm to sample from the modified posterior distribution for ***θ***. The hyperparameters that define the prior distribution for the source parameters ***θ*** ^*s*^ were exactly as described above. The prior bounds for the multipliers *m*_*J*_ were *m*_min⁡_ = 0.1 and *m*_max⁡_ = 10.0 for *J* = 1,2,…, 8 (recall that *N* = 8 in our example). The one-dimensional and two-dimensional marginal posterior distributions of the source parameters ***θ*** ^*s*^ are displayed in Figure 4. The true source parameters represented by either the solid square or solid vertical line should be compared with the best estimates of these parameters (posterior mean) marked by either a solid circle or dashed vertical line.  Table 2 summarizes the posterior mean, posterior standard deviation, and lower and upper bounds for the 95% HPD interval for the source parameters.

**Figure 5:**
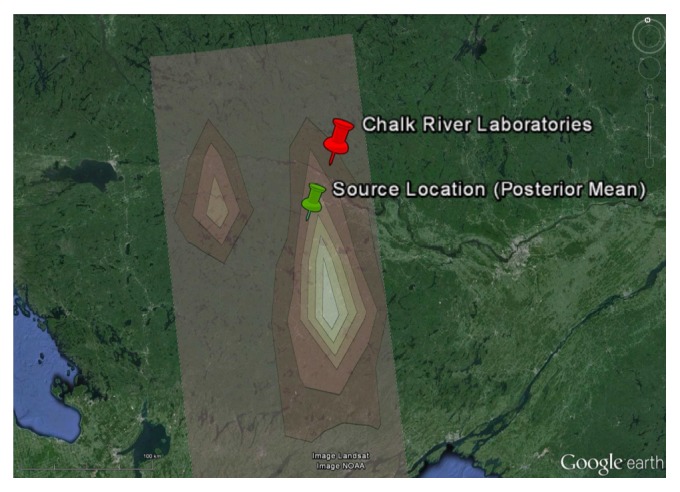
Two-dimensional marginal posterior distribution of the source location georeferenced on a Google Earth image. The marginal distribution is for the alternative measurement model which employs multipliers to compensate for the unknown model error structure.

It is clear from an examination of Figure 4 and of Table 2 that all the source parameters have been recovered with very good accuracy. More specifically, the distance between the actual source location and the best estimate (posterior mean) of the source location is about 44 km (see Figure 5), and the recovered emission rate is within 20% of the actual emission rate. Note that the accuracy in the inferred source location is roughly a factor of ten better than that obtained using the standard measurement model (which does not use multipliers to try to compensate for the unknown model errors). More importantly, the precision of the source parameter estimates (namely, the 95% credible intervals) for the alternative measurement model which utilizes multipliers to compensate for model errors contains the actual (true) values for the source parameters, in stark contrast to the reconstruction using the standard measurement model (cf. the inferred values for the source parameters in Table 1 with those in Table 2).

**Figure 6:**
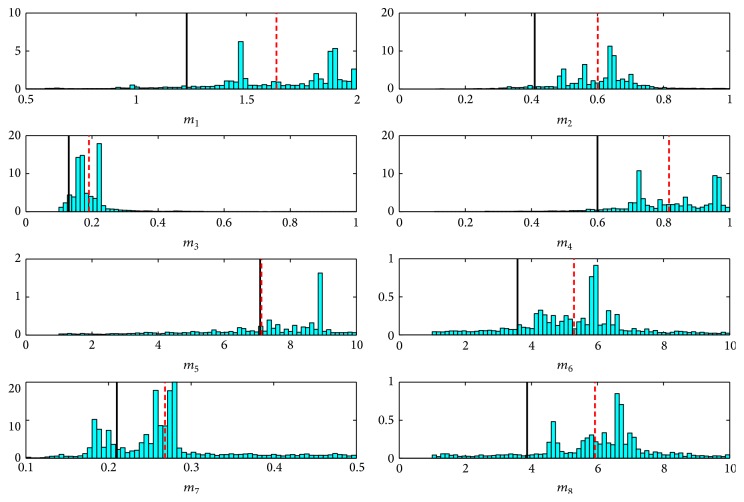
One-dimensional marginal posterior distribution of the multipliers *m*_*J*_ (*J* = 1,2,…, 8) used to compensate for the model error in the problem. The “actual” values of the multipliers are indicated with the solid vertical line which should be compared with the best estimates of the multipliers (posterior means).

In the practical real-world example of source reconstruction considered herein using activity concentration data obtained from the IMS radionuclide network, it is extremely difficult to correctly specify* a priori* both the magnitude (or scale) of the model error and the model error structure. In light of this difficulty, it is apparent from this example that incorporating multipliers (scale factors) with the predicted concentrations to attempt to compensate for the model error can improve significantly the quality of the source reconstruction. As an indication of the complexity in the model error structure for the current problem, Figure 6 exhibits the one-dimensional marginal posterior distributions for the various multipliers *m*_*J*_ (*J* = 1,2,…, 8). An examination of Figure 6 provides an indication of the complexity in the heteroskedastic model error structure, which makes it difficult to provide* a priori* estimates for the model error for use in the source reconstruction. Note that the distributions for the multipliers associated with some of the predicted concentrations are quite broad, indicating that the model errors for these predicted concentrations are not well determined. In other cases, the distributions for the multipliers (e.g., *m*_2_ and *m*_3_) are quite narrow, implying that the data contain reasonable information to constrain the model errors associated with these multipliers fairly well.

The true values for the multipliers are not known. However, we can obtain an independent estimate for the values of the multipliers in the current example because the actual source parameters are known. We can use the actual source parameters ***θ***_s_^*^ to predict the activity concentration ${\overset{-}{C}}_{J}(\theta_{s}^{*})$ that would be expected at the various observation stations. With this information, we can provide an independent point estimate for the multipliers as ${\hat{m}}_{J}=d_{J}/C_{J}(\theta_{s}^{*}),\;\;J=1,2,\ldots ,8$. These “actual” values for the multipliers are shown as the solid vertical lines in Figure 6, which should be compared with best estimates of the multipliers obtained as the mean of the posterior distributions for *m*_*J*_. A visual inspection shows that the best estimates of the multipliers are consistent with the “actual” values for the multipliers obtained from the indicated point estimates. Furthermore, the width of the posterior distribution of the various multipliers is seen to enclose the “actual” values for the multipliers. Finally, even though some of the multipliers are highly uncertain, inclusion of multipliers to compensate for the model errors does lead to significantly improved estimates for the source parameters (both in accuracy and in precision).

## 5. Conclusions

In this study, a Bayesian inferential methodology has been applied to the problem of source reconstruction for real-world activity concentration data measured by an operational network of sensors (more particularly by the IMS radionuclide network that is maintained under the auspices of the United Nations CTBT Organization). This methodology for source reconstruction has been applied to a difficult situation, namely, reconstruction of the source characteristics from the CRL release using only a small number of activity concentration measurements (8 measurements) obtained from only three sampling stations. The problem involved utilization of an operational backward LS model for long-range transport by the atmosphere on a continental scale.

The principal difficulty in the reconstruction lay in providing the correct* a priori* specification of the model error for the various predicted concentrations associated with the measured concentrations used for the source parameter recovery. A naïve specification for the model error gave a source reconstruction that was quite reasonable in terms of the accuracy in the recovery of the location and emission rate, but the precision in the estimates was generally poor in the sense that the reported uncertainty bounds in the recovery of the source parameters did not include the actual (true) values for these parameters. This led to an alternative measurement model which incorporated multipliers (scale factors) with the predicted concentrations in order to compensate for the model errors. The application of this alternative model was shown to yield significantly improved estimates for the source parameters, both in terms of their accuracy and their precision.

Although Bayesian probability theory offers a coherent and rational approach for source reconstruction, its application to real-world problems using real sensor networks and operational dispersion models will require a better understanding of both the scale and structure of the model error in the predicted concentrations. It is anticipated that the proper incorporation of information about the underlying model error in conjunction with Bayesian inference employing state-of-the-science MCMC sampling algorithms (such as the MT-DREAM_(ZS)_ algorithm) will provide the most flexible framework for source reconstruction.

## Figures and Tables

**Table 1:** The posterior mean, posterior standard deviation, and lower and upper bounds of the 95% HPD interval of the parameters *x*_*s*_ (°E), *y*_*s*_ (°N), and *Q*_*s*_ (*μ*Bq h^−1^) calculated from samples of ***θ*** drawn from the posterior distribution *p*(***θ*** | **d**, **s**, *α*, *β*, *I*).

Parameter	Mean	Standard deviation	95% HPD	Actual
*x*_*s*_ (°E)	−72.71	1.22	(−76.71, −71.56)	−77.37
*y*_*s*_ (°N)	42.51	0.32	(41.86, 43.35)	46.15
*Q*_*s*_ (*μ*Bq h^−1^)	6.68 × 10^17^	8.55 × 10^16^	(4.98, 8.33) ×10^17^	1.0 × 10^18^

**Table 2:** The posterior mean, posterior standard deviation, and lower and upper bounds of the 95% HPD interval of the parameters *x*_*s*_ (°E), *y*_*s*_ (°N), and *Q*_*s*_ (*μ*Bq h^−1^) calculated from samples of ***θ*** drawn from the posterior distribution *p*(***θ*** | **d**, **s**, *α*, *β*, *I*). The results are obtained using the alternative measurement model which employs multipliers to compensate for the unknown model error structure.

Parameter	Mean	Standard deviation	95% HPD	Actual
*x*_*s*_ (°E)	−77.66	1.04	(−79.21, −74.88)	−77.37
*y*_*s*_ (°N)	45.80	1.96	(42.69, 52.64)	46.15
*Q*_*s*_ (*μ*Bq h^−1^)	7.97 × 10^17^	1.75 × 10^17^	(6.37, 12.5) ×10^17^	1.0 × 10^18^
